# Microfluidic Device-Based Virus Detection and Quantification in Future Diagnostic Research: Lessons from the COVID-19 Pandemic

**DOI:** 10.3390/bios13100935

**Published:** 2023-10-18

**Authors:** Andres Escobar, Alex Diab-Liu, Kamaya Bosland, Chang-qing Xu

**Affiliations:** 1 School of Biomedical Engineering, McMaster University, 1280 Main Street West, Hamilton, ON L8S 4L8, Canada; cqxu@mcmaster.ca; 2 Department of Engineering Physics, McMaster University, 1280 Main Street West, Hamilton, ON L8S 4L8, Canada; diabliua@mcmaster.ca (A.D.-L.); boslank@mcmaster.ca (K.B.)

**Keywords:** microfluidics, COVID-19, diagnostics, pandemic-readiness, SARS-CoV-2

## Abstract

The global economic and healthcare crises experienced over the past three years, as a result of severe acute respiratory syndrome coronavirus 2 (SARS-CoV-2), has significantly impacted the commonplace habits of humans around the world. SARS-CoV-2, the virus responsible for the coronavirus 2019 (COVID-19) phenomenon, has contributed to the deaths of millions of people around the world. The potential diagnostic applications of microfluidic devices have previously been demonstrated to effectively detect and quasi-quantify several different well-known viruses such as human immunodeficiency virus (HIV), influenza, and SARS-CoV-2. As a result, microfluidics has been further explored as a potential alternative to our currently available rapid tests for highly virulent diseases to better combat and manage future potential outbreaks. The outbreak management during COVID-19 was initially hindered, in part, by the lack of available quantitative rapid tests capable of confirming a person’s active infectiousness status. Therefore, this review will explore the use of microfluidic technology, and more specifically RNA-based virus detection methods, as an integral part of improved diagnostic capabilities and will present methods for carrying the lessons learned from COVID-19 forward, toward improved diagnostic outcomes for future pandemic-level threats. This review will first explore the context of the COVID-19 pandemic and how diagnostic technology was shown to have required even greater advancements to keep pace with the transmission of such a highly infectious virus. Secondly, the historical significance of integrating microfluidic technology in diagnostics and how the different types of genetic-based detection methods may vary in their potential practical applications. Lastly, the review will summarize the past, present, and future potential of RNA-based virus detection/diagnosis and how it might be used to better prepare for a future pandemic.

## 1. Introduction 

The discovery of severe acute respiratory syndrome coronavirus 2 (SARS-CoV-2) in late 2019 introduced the world to its most lethal pandemic-level virus in over a century [1]. In the span of one year, the ribonuclease-based (RNA-based) coronavirus 2019 (COVID-19) grew from an epidemic-level threat, localized to only a few metropolitan cities across the globe, into the second most prevalent and lethal infectious disease worldwide [1,2]. Throughout history, highly infectious diseases, such as SARS-CoV-2, are often found to be RNA-based viruses. Unlike other more common double-stranded deoxyribonuclease-based (DNA-based) viruses such as herpes, smallpox, adenovirus, and papillomaviruses, RNA-based viruses such as hepatitis, HIV, and SARS-CoV-1 have demonstrated increased mutation rates, infectiousness, and lethality [3,4]. Many conventional diagnostic methods that do not use viral-RNA as a detection target offer some advantages such as decreased turnaround time, increased modularity, and mass-reproducibility [1,2,3,4]. However, due to the increased potential for repeated and prolonged viral evolution from an initial strain to any number of variants, the effectiveness of conventional non-RNA-based diagnostic methods for tracking and managing viral outbreaks decreases [2,3,4]. This is due to the lack of current rapid diagnostic platforms that can effectively achieve a quantitative and qualitative-based diagnosis [4,5]. Quantification-based diagnoses of viral diseases generally rely on a direct count of the target analyte, often a particular sequence of viral DNA/RNA, and report the value as the viral load [1,2,3,4,5]. The presence of any viral copies in a sample of human bodily fluid would be enough to determine positive infection but the number itself details the severity of the infection [3,4,5]. Conversely, qualitative diagnoses aim to detect byproducts associated with a recent or current infection of a particular virus [1,2,3,4]. However, those byproducts may persist beyond the active infection and have been known to provide false positive diagnoses in the past [6,7]. As a result, many conventional RNA-based detection methods such as polymerase chain reaction (PCR) and nucleic acid sequence-based amplification (NASBA) have been developed to address the need for quantification-based diagnoses. Moreover, since RNA-based methods can differentiate between variants of the same type of virus, researchers can explore the nuances and effects of different viral variants to better address the specific treatment options for each unique variant [6,7]. Through integration with other diagnostic methods, such as microfluidics, it is expected that RNA-based virus diagnostic technology will surpass the qualitative and quantitative capabilities of non-RNA-based detection methods [5,6,7].

SARS-CoV-2 was known to have mutated into several relevant variants over the course of the COVID-19 pandemic, each with varying symptom phenotype and symptom severity across individuals infected with identical variants [8]. In addition, it has been shown that even across multiple variants of the same parent virus, the variance in the development and onset of symptoms in RNA-based viruses greatly differs between strains and becomes increasingly difficult to accurately diagnose through conventional non-genetic diagnostic methods. Thus, RNA-based detection methods such as PCR and NASBA are required to effectively differentiate between variants of certain highly infectious RNA-based viruses to provide effective treatment and outbreak management options against the consequences of a pandemic-level threat [9]. To accomplish such tasks, earlier iterations of RNA-based detection methods required expensive machinery to effectively analyze and track patient samples in a timely manner. However, the nature of delayed symptom-onset in certain RNA-based viruses presented several significant issues to diagnostic efficacy in previously established conventional methods [10]. With the delayed onset of SARS-CoV-2 symptoms in infected individuals being roughly 7–12 days, after initial exposure, an infected individual may only present mild or common flu-like symptoms that would delay the need for testing with sophisticated RNA-based techniques [11]. In some cases, infected humans would not even present symptoms at all throughout the entirety of their infectious period [10,11,12]. Moreover, due to the lack of access to these more sophisticated RNA-based techniques for diagnosing RNA-based viruses as a result of limited trained personnel and limited numbers of specialized facilities to process patient samples, RNA-based techniques were not previously effective in determining the infectious status of an individual [13]. Thus, the need for improved diagnostic methods to assess the infectious status of people with RNA-based viral infections more accurately and quickly is evident and should be focused upon to address the inherent limitations in conventional RNA-based diagnostic assays. Several commonly observed limitations in conventional RNA-based diagnostic techniques can be readily addressed through microfluidic integration [14].

Given the highly infectious nature of the RNA-based SARS-CoV-2 virus, its ability to evolve into newer and more transmissible variants, as well as its ability to cause the development of varying symptoms, makes COVID-19 one of the most lethal and difficult-to-manage viruses the world has seen [15]. With at least 6.2 million global deaths officially caused by SARS-CoV-2 (since December 2019), SARS-CoV-2 and its numerous variants will likely continue to plague our society for many years to come [14,15,16]. The shortened lifespan and increased evolutionary rate of RNA-based viruses pose problems to detection methods often utilized in qualitative-exclusive diagnostic tools. As a result, diagnostic tests with very small turnaround time and short production times were quickly prioritized for research and development. Qualitative diagnostic tests such as the rapid antigen tests developed throughout the first two years of the pandemic have allowed many people to diagnose themselves and slow down the progression and continued evolution of SARS-CoV-2 variants more readily but did not stop it [17]. As a result, many people in more resource-wealthy countries around the world have had an increased chance of returning to some sense of normalcy in their daily lives [18]. However, access to this luxury cannot be readily afforded by resource-limited countries and people, thus putting the societies that do not have ample supplies and access to these diagnostic tools at greater risk for the continued development and spread of SARS-CoV-2 and its variants [19]. Therefore, the importance of affordable diagnostic tools with both qualitative and quantitative capabilities cannot be understated and must be explored to eventually overcome the limitations inherent to exclusively qualitative tests. As will be explained below, the integration of microfluidic technology offers the ability for conventional qualitative diagnostic tools to be a more modular, more portable, more affordable, and more accurate means of diagnosing RNA-based viruses [20]. This review will outline the historical significance of microfluidic research and its relevance to RNA-based microfluidic assays and justify the need for continued microfluidic research to better prepare for future pandemic-level threats.

## 2. Integration of Microfluidic Technology throughout History

**Figure 1:**
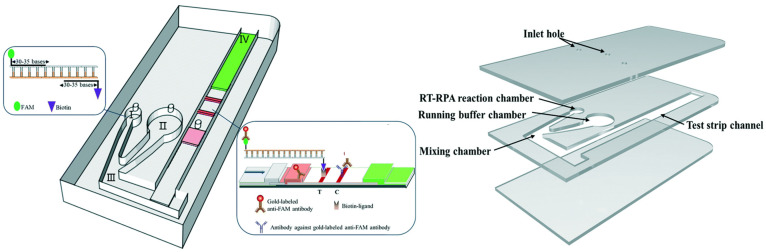
A schematic diagram of a microfluidic device, integrated with RNA-based detection, for SARS-CoV-2 diagnosis. Adapted from [28].

Microfluidic devices harness the microscopic-specific properties of fluids to facilitate the performance of specialized tasks, such as diagnosing viral infections [21]. These devices commonly exist as a combination of biocompatible polymers and substrates, with several microchannels linking inlet and outlet ports for fluid flow [22]. As seen in Figure 1, the coupling of these ports is directed by a system of microchannels, a system of narrow tubes with microscopic hydraulic diameters to facilitate laminar flow, and a state of fluid dynamics where no lateral mixing occurs. Normally, at higher velocities, two or more liquids at macroscale volumes flowing through channels together would initiate lateral mixing [23]. This phenomenon is typically unideal for fluid-dynamic research as lateral mixing often leads to turbulent flow and chaotic mixing, which can cause variable results [22,23,24,25]. By facilitating the movement of micro-scale fluids through microchannels without lateral mixing, also known as laminar flow, more reproducible and significant results can be achieved [26]. Microfluidic devices can readily employ laminar flow due to the differences in fluid properties between micro- and macro-scale volumes, allowing for a dramatic increase in control and precision to relevant experiments [27]. The history of microfluidic technology extends as far back as the early 1900s but has only recently seen increased use in parallel to technological advancements [16]. Subsequently, the successful integration of microfluidics into diagnostic technology has facilitated a wealth of novel technological innovations that help to overcome limitations in conventional techniques, as seen in Figure 1.

Over the past century, the seemingly unlimited number of impactful applications for microfluidic technology has been seen across various industries such as printing technology, microelectronics, microbiology, and diagnostics [29]. In the 1950s, one of the first major uses for early-stage microfluidic technology was shown to have been effectively implemented in inkjet printing. By harnessing the imperceptible size and innovative designs of microchannels, the printing industry and researchers developed a preliminary means to control the release of small volumes of fluids in an extremely precise manner, something no other technology was able to at the time [29]. Facilitated by the precision of early-stage microfluidics, inkjet printers revolutionized the industry to the point where it is still commonplace today and led to the applications of microfluidic technology in other fields, such as microelectronics.

During the 1960s to the 1980s, the first recorded uses of microfluidic technology in the microelectronics industry had been employed through microfluidic-based semiconductor processing technology to develop devices known as micro electromechanical systems (MEMS) [30]. As an adaptation of microfluidic technology used in the development and advancements in inkjet printer technology, techniques such as photolithography and deep silicon etching are just two examples of microfluidic-based semiconductor processing methods that have facilitated the large technological advancements provided by MEMS devices [31,32,33]. MEMS are micro-sized machines that integrate mechanical and electrical components to create tiny digitally integrated devices or systems which can be used for numerous applications. Prior to the development of MEMS devices, many previously large-scale computational processing or digital controllers required large amounts of expensive resource-intensive equipment to operate [34]. By building upon the microfluidic technology incorporated in early-stage inkjet printers, the microelectronics industry has opened the world to novel technological advances only possible through devices integrated with MEMS sensors and other related apparatus [35]. Some significant microfluidic applications of MEMS devices include MEMS inkjet printheads, digital control of microfluidic devices, medical devices, and, most importantly, MEMS sensors.

In the last 30 years, microfluidics has also expanded into the microbiology and diagnostic industries, greatly improving the automation capabilities of conventionally laborious work such as cell culturing and drug testing [1,18,19,20]. Traditionally, cell culturing involved tedious and time-consuming human-driven processes in laboratories where cell cultures and their storage containers are prone to decreased reproducibility and significance in results due to increased exposure to external environmental factors [36]. Cell culturing is an integral microbiological technique used in several different ways to aid in evolutionary studies, genetic studies, and more. To address the decreased reproducibility and increased variability in these cell culturing techniques, microfluidic technology has been used to miniaturize previously existing culture systems to achieve a controlled fluid microenvironment. The development of these controlled microenvironments can be achieved by microfluidic-based methods such as soft lithography and photolithography [37]. Soft-lithography, similar to photolithography, allows for precise etching of a design onto a substrate, which in turn, will allow for the development of numerous reproducible and precise microfluidic devices [38]. Large-scale production of these precise microenvironments is an integral component of more significant applications of microfluidic technology, namely, organ-on-a-chip and diagnostic technology.

Microfluidic technology can be used to facilitate in vivo human organ responses to drug testing by aiding in the production of biocompatible microfluidic platforms where specific organ tissues can be mimicked [39]. Known as organs- or tissues-on-a-chip, these devices have proven to be invaluable in modern medical research as they provide researchers with relevant experimental data on humans without any ethical dilemmas. Organ-on-a-chip technology has been highly researched in recent years as scientists work towards a human-on-a-chip, which could mimic the physiological responses that a complete human organ system would produce in following a certain modification [40].

**Figure 2:**
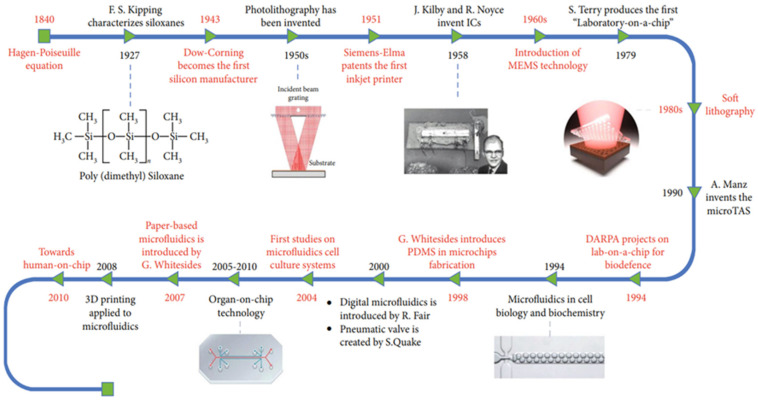
Summary of the historical advancements of microfluidic technology over the years. Reprinted from [41].

Figure 2 highlights several significant past and present-day applications of microfluidic technology in various fields. The advancements in microfluidic technology over the past several decades have demonstrated the seemingly unlimited potential of microfluidic technology. Microfluidic technology’s contribution to fields such as microelectronics, printing, and drug-based diagnostics are significant and varied but are by no means the most significant potential application of microfluidic devices [41]. Since the COVID-19 pandemic, the need for rapid and diagnostic platforms has been considered an essential resource in combatting the continued and future potential spread of highly infectious diseases. However, the initial limitations of mass-producing an effective on-site rapid diagnostic tool, such as supply and distribution logistics, severely hindered the effective outbreak management of SARS-CoV-2 [42]. With the continued presence of viral-RNA variants, previously established non-genetic (indirect) diagnostic tools struggled to keep up with an ever-increasing demand for effective rapid testing. Thus, the improved diagnostic potential of RNA-based (direct) microfluidic devices was explored as a potential solution.

## 3. Microfluidic Technology as a Diagnostic Tool for RNA-Based Viruses

Many different well-known diseases such as hepatitis C, influenza, and Middle East respiratory syndrome (MERS) are caused by viral DNA or RNA and can be further subdivided into many subspecies [42,43]. In recent history, coronaviruses like MERS have been highlighted due to their increased lethality and infectiousness, compared to other more common RNA-based viruses such as seasonal influenza [43,44]. Conversely, well-known DNA viruses such as herpes and poxvirus are less problematic due to their apparent lowered rate of lethality compared to RNA-based viruses such as SARS-CoV-2 [43,44,45]. Coronaviruses, such as SARS-CoV-2 and MERS, are zoonotic RNA-based viruses that can readily infect both animals and humans by attacking the respiratory system, and potentially harming other vital organs as a consequence of the overburdened immune response [45]. With the numerous amounts of SARS-CoV-2 variants emerging over the course of the COVID-19 pandemic, newer strains and newer consequences to humans infected with SARS-CoV-2 will continue to evolve. Therefore, the need for faster and more accurate RNA-based diagnostic techniques is required to help healthcare professionals and society consider improved isolation practices and better-personalized care earlier. Earlier treatment of patients infected with SARS-CoV-2 has been shown to greatly improve the outcome during and after the infectious period [46]. Current large-scale RNA-based virus diagnostic methods, with no microfluidic integration, suffer from several limitations inherent to the detection and quantification methods used to facilitate the diagnosis [45,46]. The increased turnaround time, the need for trained operators, expensive equipment, and accessibility are examples of limitations that hinder non-microfluidic-based diagnostic methods from demonstrating their full potential. Thus, the challenges inherent to conventional RNA-based virus diagnostic methods must continue to be overcome and microfluidic integration is the best means to accomplish this, as will be explained below.

### 3.1. Conventional and Emerging Non-Microfluidic Diagnostic Methods

To best understand the potential advantages of microfluidic integration we must first explore the limitations seen in conventional RNA-based diagnostics and how microfluidic technology can address those limitations. Early and more reliable diagnosis of viral diseases, such as SARS-CoV-2, can lead to personalized and improved treatment outcomes [47]. Currently, rapid immunohistochemistry tests are the gold standard in on-site and point-of-care (POC) viral diagnostics. These rapid diagnostic tests are a form of indirect viral detection as they employ designed biological probes to target targets in the patient sample that are expected to be produced during a positive infection [48]. This technique, however, is limited in its ability to analyze patient samples as it can only act as a qualitative test of viral infection once a patient’s viral load reaches a certain threshold [41,44,48]. Alternatively, there are tests that exist which can achieve both quantitative and qualitative analysis; however, they are often limited to genetic based amplification of the viral genome. The two most important conventional direct detection methods capable of both quantitative and qualitative analysis are nucleic acid-based amplification tests (NABAs) and real-time polymerase chain reaction (RT-PCR) [49]. Some advantages of these direct detection methods include the possibility to detect several viruses simultaneously and increased selectivity and detection limit, but in turn, sacrificing speed, cost, and ease of access. This lends itself to the use of a derivative of RT-PCR, known as quantitative RT-PCR (qRT-PCR), as a gold standard for the off-site detection of SARS-CoV-2 when a laboratory or research center is available [50]. This adapted version does, however, suffer from the same limitations in cost and time but also is not nearly as sensitive as it should be [51]. Therefore, other amplification-based methods are being explored as well. As a result of the continued shortage of equipment, staff, and resources in healthcare centers, such as hospitals, prevent ideal disease management, and treatment, a more accessible, affordable, and rapid method of diagnosis is thus needed.

Microfluidic devices must be looked at as the future gold standard in rapid diagnostic testing as they may offer cheaper, faster, and more convenient testing to patients in any setting. By facilitating the increased speed and convenience in the diagnostic process, microfluidics can aid in improving treatment outcomes of infected patients, likely lowering the death rate. The ability to use microfluidic integration to cross several detection methods further cements its diagnostic potential in future development.

### 3.2. Advantages of Microfluidic Integration

Microfluidic integration does not require a standalone microfluidic platform that utilizes its own unique detection method to be effective but can instead be used to improve upon most currently existing detection methods by simply facilitating the miniaturization of those methods, in a highly controlled manner [52]. More specifically, microfluidic devices can more readily be outfitted with one of several different functional detection modules to best assist in diagnosing the target of interest. For example, a base microfluidic device, such as the one seen in Figure 1, can be outfitted with an optical waveguide to measure the number of cells passing through the “laminar flow channel” portion of the device [53]. This functional modification would in turn add a degree of quantitative measurements previously unattainable without microfluidic integration. Microfluidic devices can therefore impart various advantages to the detection method being used, through flexibility in substrate type selection, modularity in channel design, portability, and their sample processing capabilities.

As seen in Table 1, to facilitate a better understanding of the myriad of uses and advantages that microfluidic integration offers, understanding the different classifications of microfluidic devices is essential.

### 3.3. Flexibility of Substrate Type Selection

Substrate types define the material of each microfluidic device in which the reactions take place, such as polymers or glass [54]. Therefore, classifications based on the substate type are useful for better understanding the limits of those substrates and their respective purposes. Substrates can be composed of various types of materials that dictate the physiochemical foundation of the reaction. The five most prominent substrate materials include silicon, glass, polymers, paper, and ceramics, each with their own advantages and limitations [55]. Historically, as the first substrates used in microfluidic devices, silicon and glass were chosen because of their inherent mechanical strength, chemical resistance, and favorable electrical properties [54,55]. However, the rigidity of these materials as well as their high fabrication costs greatly reduced their practical applications. At the turn of the millennium, polymer-based microfluidic devices succeeded silicon and glass as the most common substrate. Their biocompatible nature and low fabrication costs directly addressed the limitations of their predecessors while maintaining their favorable traits [54,55]. Similarly, paper-based microfluidics are ideal for mass production because of their simple composition and abundance in nature [54,55]. The sole downside to paper substrates is the convoluted process required to engrave the microchannels into the device. Interestingly, ceramic-based microfluidic devices have begun to rise in popularity due to their diverse properties. Ceramic substrates are extremely versatile, with no glaring limitations and almost all have relatively good mechanical strength, low cost, and favorable electrical properties. In every case, there is a substrate with at least one unique advantage which cannot be readily circumvented by non-microfluidic diagnostic devices. Thus, the modularity material choice can offer is a tenant of microfluidic technology’s potential.

### 3.4. Improved Channel-design Development and Testing

**Figure 3:**
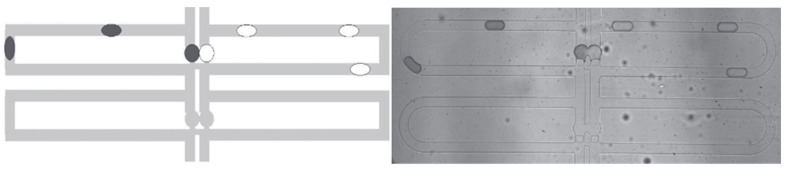
A comparison between software models of hopeful microfluidic device designs and their resulting implementations; channel widths are 25 µm. Adapted from [58].

Conventional, non-microfluidic, RNA-based diagnostic devices are normally limited to one desired function during each test [56]. However, the functionality of an integrated microfluidic device is primarily dictated by the ingenuity behind the nature of their channel design. As a result, the channel design can further expand the diagnostic potential of microfluidic devices by being optimized into one of two main types of categories: continuous and droplet-based microfluidics [57]. Through highly sophisticated computer-based simulations, researchers can now model their channel designs before physically building them and investigate the effects of their design on the fluid dynamics of the microfluidic system. As seen in Figure 3, the left image represents a snapshot of a computer-generated channel design compared to the experimental product after initial modeling. With improved modeling technology, the number of unique channel designs is seemingly infinite, and their diagnostic potential can be greatly improved.

Fluid-mixing is a phenomenon required to instigate several important bioreactions in the field of diagnostics. Bioreactions such as PCR-amplification, fluorescent probe detection, and immunohistochemical detection all rely greatly on the effectiveness of the reagents to reach and bind to their respective targets of interest [57]. When biological reagents are added to a human biological sample, mixing is an essential process in ensuring that the reagents can be evenly distributed within the sample to increase the binding efficiency. In the case of macroscopic fluids, this mixing can only occur through turbulent flow chaotic mixing, otherwise known as turbulent flow [59]. As a result, turbulent flow mixing leaves no control over the lateral mixing of liquids as they travel through the channels [59]. However, as previously stated, microfluidic devices operate on the principle that they can readily facilitate laminar flow. Laminar flow prevents uncontrollable and undesired lateral mixing of fluids, passing through channels, to allow for the efficient and controlled transport of fluids throughout a system [59]. In most conventional diagnostic applications, due to the highly sensitive nature of the mixing in diagnostic tests, trained personnel are required to operate expensive equipment and handle relatively large amounts of expensive reagents. Due to this requirement in conventional diagnostic techniques, fiscal limitations on the development and production of fast and mass-producible diagnostic tests hinder the practicality of implementation. Previously established conventional diagnostic techniques often utilized DNA/RNA amplification to facilitate the combination of any number of these essential properties often seen in large-scale sample processing. Historically, large-scale sample processing was almost exclusively limited to laboratory and industrial spaces as the cost of operating the equipment and safely handling the samples was high [60]. Amplification-based methods, better known as direct detection methods, have seen many advancements and various forms over the years such as PCR, quantitative PCR (qPCR), reverse transcriptase PCR (RT-PCR), loop-mediated isothermal amplification (LAMP), and CRISPR-Cas amplification methods [60]. Though these amplification methods offered the ability for simultaneous qualitative and quantitative analysis, the turnaround time, reagent cost, and logistical costs of having trained operators processing those samples in a lab severely limited direct detection’s practicality for diagnostic detection. In contrast, amplification-free (indirect) detection methods, often referred to as immunoassay-based detection methods, overcame the time limitation unique to amplification-based methods but often still required sample processing in a lab. Examples of well-known conventional indirect detection methods adapted for diagnostic uses include enzyme-linked immunosorbent assay (ELISA), lateral-flow assays, and blotting techniques [61]. In both cases, amplification-based or amplification-free detection methods, significant turnaround times and logistical and production costs limited the mass production and feasibility of these detection methods in mass production. In essence, the extended turnaround times in non-microfluidic-based diagnostic devices may have contributed to the increased infectiousness and spread of SARS-CoV-2 prior to the increase in accessibility of rapid tests. Although rapid tests managed to help curb the spread of SARS-CoV-2 with its increased turnaround time, the inability for quantitative analysis and the potential for false negatives hinder the long-term effectiveness of rapid tests [61]. Therefore, one of the most significant advancements in microfluidic-based diagnostic technology is the ability to process tests without the need for laboratory-grade equipment or expensive logistical costs. By increasing the modularity and portability of diagnostic methods, microfluidic devices have demonstrated their potential for continuously overcoming previous technological limitations in the hopes of producing increasingly optimized diagnostic devices for on-site or POC detection. With fewer costs required for trained operators, expensive laboratory equipment, and improved robustness, more resources might be able to be put back into further optimizing the capabilities of microfluidic technology in diagnostic devices. For instance, one of the main shortcomings of non-microfluidic PCR diagnostics is its extended turnaround times due to the equipment requirements to effectively analyze and record the processed samples [61]. Integrated microfluidic devices were able to directly overcome this obstacle by facilitating the miniaturization of this platform in such a way that the reliable mass production of RT-PCR chips was possible. Whether it is through direct or indirect means, the analysis of RNA-based viruses can be greatly facilitated through the ability of microfluidic devices to simultaneously process and analyze several targets in one individual test. Therefore, by facilitating these different forms of mixing under laminar flow, microfluidic devices offer several different means to increase control and reproducibility in diagnostic devices, while reducing operational and processing costs.

**Figure 4:**
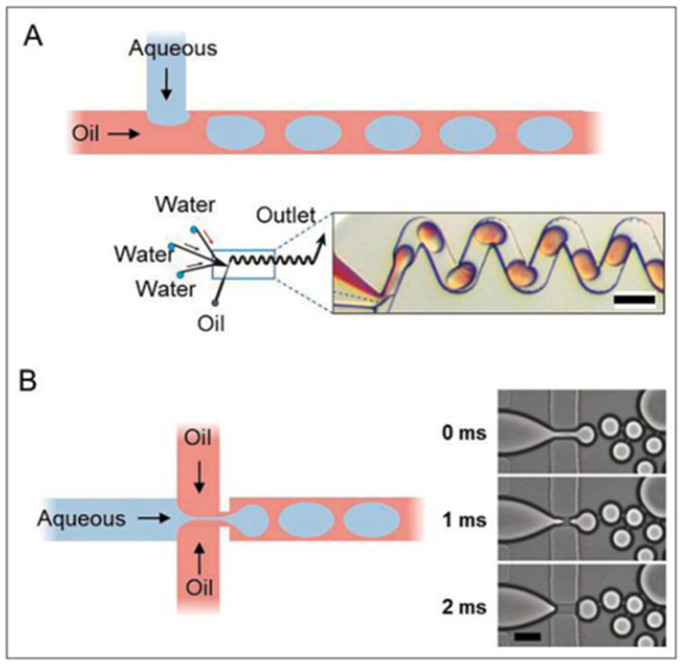
(**A**) demonstrates one of the most popular microfluidic design shapes, a “T-junction”, usually for generation of slower-moving and larger droplets. (**B**) demonstrates a “cross-junction” that can facilitate the formation of droplets in high-throughput devices. Scale bars represent 100 µm. Reprinted from [64].

Continuous microfluidic devices can be employed to create homogeneous mixtures at varying concentration levels of each fluid [62]. Liquids of comparable viscosities are pumped through their respective microchannels and funneled into one junction. At the junction, laminar flow prevents the fluids from mixing laterally but the dozens of alternating layers of each liquid upon the other give the resulting solution the appearance of a homogeneous mixture [62]. As a result, continuous microfluidics more closely resemble their macroscopic counterparts in the sense that they mix intuitively but in a much more controlled and reproducible manner. By altering properties such as the initial rate of flow, channel dimensions, and overall channel design, the concentration of a particular fluid within this mixture can be easily manipulated. Concentration manipulation offered by continuous-based microfluidic devices has facilitated advancements in diagnostic potential for their ability to control and self-contain key bioreactions that require mixing into homogeneous mixtures. For example, PCR reactions used to be limited to use with trained technicians, expensive thermocyclers, and reagents, making them very limited in their on-site potential [63]. As seen in Figure 4, through the integration of continuous-based microfluidics, many of these PCR reactions can now be performed without the need for trained technicians. Despite the numerous advantages that continuous-based microfluidics offers, it carries the inherent risk that the resulting solution will contain uneven distributions of the independent fluids as there is a certain aspect of probability that dictates how the fluids mix. This potential mixing issue is characteristic of continuous microfluidic devices but can be overcome using droplet microfluidic devices.

Contrary to continuous-based microfluidics, droplet microfluidics allow researchers to exploit the microscopic shear forces produced by the interaction of two or more analyte fluids, with contrasting viscosities, to create numerous controlled micro-scale droplets of comparable size [65]. The fluids flow through distinct microchannels towards a junction where a mixture of one or more fluids of comparable viscosities becomes encapsulated by another significantly more viscous fluid, such as oil, known as the sheath fluid. The shear flow forces applied on the analyte mixture by the sheath fluid are sufficient to sever a drop and allow it to proceed through the junction. Ideally, this process occurs at regular intervals and is funneled towards the outlet port where the final heterogeneous solution may be collected and processed [66,67]. Through droplet-based microfluidics, researchers can ensure that each droplet of the desired liquid contains sufficient reactants to perform the intended reaction in a highly controlled manner. Moreover, the ability to produce hundreds or thousands of these comparable microscopic droplets allows droplet microfluidic devices to facilitate high throughput processing. Due to the highly modular nature of droplet microfluidics, there are three significant sub-categories: ultrahigh throughput, controlled, and digital microfluidics. Each subcategory is designed to focus on providing a significant advantage in one main characteristic of the design but can be designed to implement advantages of more than one of these characteristics [68]. Therefore, droplet microfluidics have the potential to process high throughput biological assays much faster and more controlled compared to continuous-based microfluidic devices.

Both continuous and droplet microfluidic channel designs have been used in several iterations of RNA-based assays and have all improved upon the inherent diagnostic capabilities of the detection methods used [2]. Continuous-flow channels allow for increased efficiency in both lateral and vertical mixing, which can be used to improve the likeliness for a bioreaction in micro-scale volumes to occur. In turn, droplet microfluidics further improve upon the benefits of continuous-flow microfluidics by facilitating the mass production of hundreds or thousands of discrete microvolume bioreactions that can greatly improve the sensitivity, specificity, and throughput of a diagnostic test. Channel designs and material type do not, however, account for all the advantages microfluidic integration has to offer. Portability, decreased turnaround time, and processing capacities are also important benefits to microfluidic technology.

### 3.5. Multiplexing Advantages of Microfluidic Integration

The historical standard for being able to process and target diagnostic samples for one biological target of interest per test, better known as singleplexing, has endured several decades of technological advancements and remains quite commonplace today [68]. With recent improvements in digital technology, this limitation may no longer continue to remain commonplace. The ability for tests to process and target more than one target of interest, per test, is better known as multiplexing. Multiplexing in conventional non-microfluidic diagnostic technologies is intrinsically difficult due to the need for a balance between specificity and selectivity in multiple targets [68]. Due to a lack of modularity in tests that can only analyze one target of interest, also known as singleplexing, many non-microfluidic diagnostic devices have been unable to capture as much data as possible in each tested patient sample. Moreover, as previously discussed, many conventional methods with increased specificity and selectivity must sacrifice portability and decreased turnaround times [69]. Over time, through microfluidic integration, the limitations on processing one target per test have been shown to be far less of an issue. In essence, the integration of microfluidics gave rise to the ability for biological assays, especially RNA-based assays, to be multiplex in nature. Multiplex devices are unique in their ability to detect and analyze numerous analytes simultaneously, while facilitating greater efficiency and accuracy through integration with microfluidics. Through multiplexing, one sample can be separated and processed for two or more targets of interest, maximizing the research and diagnostic potential in each test [69]. This directly contrasts with conventional diagnostic technology that was only capable of processing one target of interest per test, otherwise known as singleplex assays. In the early-stages of the COVID-19 pandemic, there was much confusion and uncertainty surrounding the nature and symptoms associated with SARS-CoV-2. Given that there are hundreds of viruses capable of infecting humans, there are bound to be symptoms shared amongst different viruses, making superficial diagnosis nearly impossible [70]. Therefore, testing for a combination of multiple targets to improve specificity is a necessary feature for optimizing diagnostic technology. For instance, SARS-CoV-2 patients sharing similarities in their symptoms to seasonal-influenza infections had initially led to several false diagnoses in the early stages of the COVID-19 pandemic. This left many people being treated incorrectly for a more severe infection than required, with potentially negative consequences, and left many people infected with SARS-CoV-2 undertreated. Without first identifying biological targets that can specifically differentiate between the two, patients were not being accurately diagnosed, resulting in a mismatch of necessary treatment that could pose both short-term and long-term harm to the patients [70]. Moreover, given that certain viruses can evolve or mutate into numerous variants or subvariants that share common biomarkers, the complexity required to accurately diagnose infected patients significantly increases [71]. Thus, the need to accurately test for increasingly complex combinations of biomarkers specific to certain diseases, and their respective subtypes, is evident. Through microfluidic integration, multiplexing is possible and could be used to determine the exact type of virus or disease based on a combination of biomarkers specific to one strain. Equipped with this knowledge, healthcare professionals, governments, and industry alike, can better address the needs of the patient based on the data specific to that strand, contributing to the importance of microfluidic integration in diagnostic technology. The classification of diagnostic technologies as singleplex or multiplex will be important in our future effort to improve our diagnostic capabilities. 

### 3.6. Summary of Advantages Due to Microfluidic Integration

**Figure 5:**
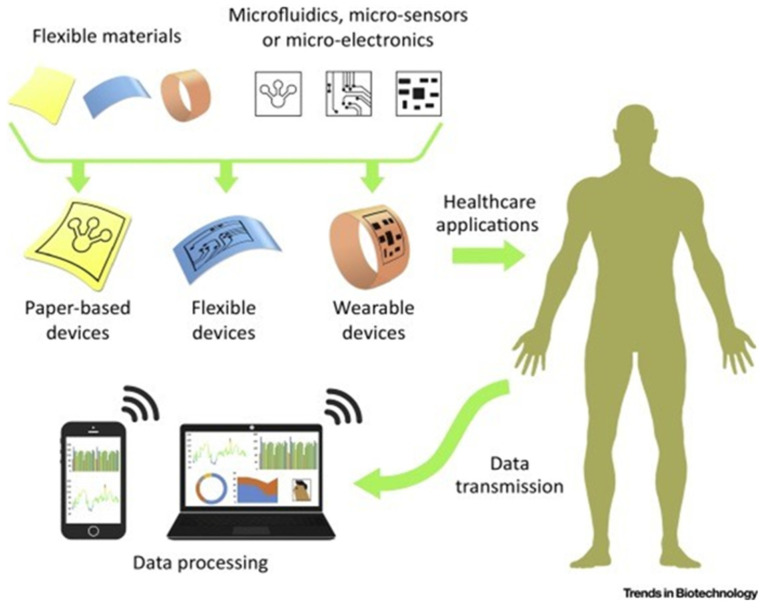
A schematic for what an idealized rapid and on-site microfluidic device can offer the field of diagnostics and those devices’ basic components. Reprinted from [71].

Lastly, the most important feature to any diagnostic device lies in its accessibility, and by extension, its potential for on-site or point-of-care analysis [72]. On-site diagnostic devices are capable of being transported, used, and processed all in one location, without the need for large-scale equipment or trained technicians. Point-of-care diagnostic devices are similar to on-site devices in terms of their portability and modular testing capabilities but differ in their need for processing with expensive machinery and equipment that may be more difficult to transport [55,61,71]. Traditionally, portable RNA virus testing was confined to off-site labs. Consequently, patients remained unaware of their infection status for days after their diagnostic test, potentially contributing to the further transmission of the virus. Point-of-care testing directly addresses this complication, with the diagnostic analysis occurring at the point of care immediately after the diagnostic test, leading to significantly reduced turnaround times [15]. Point-of-care testing was further refined into on-site testing, which can utilize rapid and quantitative diagnostics in resource-limited environments, allowing for near instantaneous results for the patient [1,33,34,35,36]. Therefore, an effective diagnostic device should be designed and mass-produced to be more portable, reproducible, and processible without the need for expensive equipment. By combining all the attributes and advantages of microfluidic integration, researchers and industry have been able to produce incredibly complex and efficient diagnostic tools, as seen in Figure 5. It is only through the modular nature of microfluidic technology that increased portability, affordability, robustness, and digital integration can be achieved. More importantly, microfluidic technology might be able provide more accurate diagnoses of patients earlier and hopefully improve patient treatment outcomes and severity in the event of another massive viral outbreak.

## 4. RNA Virus Diagnostics: Past, Present, and Future 

Microfluidic devices are extremely versatile tools with great potential, but they are not without their limitations. The micron-level precision required to effectively assemble this technology historically resulted in extremely high fabrication costs for each device [1,56,57,58]. Prior to the age of 3D-printing technology and high-performance modeling software, the mass production of microfluidic-based diagnostic devices could have never been practical for marketing to the public [70]. Despite the technological advancements achieved since its conception, the financial burdens of microfluidic technology still outweigh its benefits. As such, the inherent cost of microfluidics remains the largest barrier in the prevention of their mass production and status as the gold standard for the detection and quantification of RNA-based viruses.

### 4.1. Past RNA Diagnostics 

Modern-day microfluidic technology has evolved tremendously over the last several decades, serving various functions and spanning multiple industries. Although microfluidic technology has demonstrated its historical significance across many fields, its most relevant function in contemporary society lies in its diagnostic applications [14,15,16,17,18]. Several key properties of contemporary microfluidic devices originated from the industries for which microfluidics were originally designed, including molecular analysis, biodefense, molecular biology, and microelectronics. In the early 20th century, novel viral diagnostic techniques were in high demand [21,22,23,24]. With the seemingly infinite amount of RNA virus types and features, researchers found themselves with a plethora of potential methods and biomarkers to employ in the detection of viruses.

RT-PCR-based assays have historically dominated the RNA diagnostic market to such an extent that they were considered, up until the last decade, as the “gold standard” [1,23]. This off-site diagnostic technique employs reverse-transcription and PCR amplification on RNA, in patient samples, to determine the presence of target viral genomes. This method was considered the gold standard for its ability to assess patient samples quantitatively, qualitatively, and off-site to provide an accurate wealth of information related to the active infectiousness of patients. In times where the turnaround times and sensitivity of this previous gold standard led to results which may not have been time-sensitive, as in the COVID-19 pandemic, extended turnaround times and off-site processing were not necessarily significant limitations [73]. Moreover, despite having its accuracy being called into question on several occasions, RT-PCR-based assays such as qRT-PCR remained the primary RNA virus diagnostic method over the years. The gold-standard status maintained by RT-PCR-based assays would, however, not be the only detection method being explored, prior to the advent of popularized microfluidic technology in diagnostics in the 2010s. Around this time, research into alternative diagnostic assays that might have been able to overcome several limitations presented by RT-PCR-based assays was being performed. Among them, CRISPR-based diagnostic assays have emerged as one of the most promising substitutes for RT-qPCR [62,63].

CRISPR-based assays offer an alternative amplification-based direct detection method to target nucleic acid biomarkers, providing them with the potential to be used as on-site technology [74]. CRISPR-based methods utilize a Cas-protein and guide RNA complex that facilitate highly specific genetic cuts. By designing a guide RNA with significant homology to the target RNA or DNA, researchers were able to essentially manufacture systems that can target very specific sequences of RNA in viruses to uniquely identify them [73,74]. By adding genetic probes to these CRISPR systems, researchers were able to add a new level of detection and quantification to this emerging diagnostic method. However, traditional CRISPR-based methods can often be lengthy and labor-intensive due to the numerous procedures involved in predicating the analysis, including nucleic acid extraction and amplification. Moreover, the likelihood of false-positive results prone in the traditional tests reduced the viability of this method as a reliable diagnostic method [15,71,74]. While nucleic acid-based detection is exceptionally sensitive and precise, its drawbacks include highly trained staff, high costs, and the possibility of carry over contamination. Nevertheless, nucleic acid-based detection techniques remain prominent in scientific research, with one of its primary applications being determining links between certain viruses and other conditions. With turnaround time and accuracy often being cited as the main flaws in RNA amplification-based viral detection methods, the expansion into immunochemical diagnostics was a hopeful alternative.

Without the need for amplification or genetic editing, immunological detection assays have always experienced a lowered cost and much more modularity. These immunological assays are often robust, portable, and easier for untrained users to facilitate without a professional. Immunoassays such as enzyme-linked immunosorbent assay (ELISA), Western Blot, and Immunostaining employ antibodies to detect and target specific virus-associated antigens using cell culture methods [75]. Some of these historically significant viral diagnostic methods and their discoveries are noted in Table 2. While these methods are cost-efficient and robust, the extended turnaround times as well as lowered sensitivity and accuracy relative to nucleic acid-based detection are unideal for mass production and the effective containment of a virus. 

Both nucleic acid-based and immunological diagnostic techniques have their respective flaws and limitations. However, unlike indirect detection, which requires a certain pre-existing amount of virus propagation, direct detection may be favorable due to its ability to diagnose viruses in early stages of infection, preventing further transmission. As such, RNA-based virus diagnostics have primarily revolved around direct virus detection through history as the prevention of transmission and effective containment of the virus are of extremely high importance in virus detection. 

### 4.2. Current Status of RNA-Virus Diagnostic Microfluidic Technology 

Throughout the COVID-19 pandemic, the exposed shortcomings and limitations of current diagnostic devices incurred severe socioeconomic repercussions worldwide [25]. These traditional diagnostic devices had low accuracy (false negatives and false positives), poor portability, instrument dependency, and labor intensity which led to a lot of confusion and the continuous spread of disease [1,25]. Microfluidic diagnostic devices that are currently being researched have demonstrated the ability to overcome many of these limitations. Therefore, a summary of some of the most relevant diagnostic technologies, integrated with microfluidics, is discussed in this section.

Several notably significant detection methods have been adapted with microfluidic technology to augment their diagnostic properties. As seen in Table 3, properties used in recent SARS-CoV-2 diagnostic methods such as the limit of detection, turnaround time, and sample volume sizes have reached novel heights. Diagnostic capabilities of table-top diagnostic methods such as those listed in Table 2 show great future diagnostic promise as turnaround times and reduced costs would greatly increase the validity and potential of these conventional diagnostic methods as on-site or POC devices [76]. Currently, there are many promising examples of these microfluidic POC devices for RNA-based diagnostics showing great improvement in several of the diagnostic qualities. For example, newer ultra-fast RT-PCR assays offer a portable SARS-CoV-2 detection platform that can detect as few as 20 viral copies per test and 10 (10 µL) samples per run, in under 40 min [1,41,51]. This level of parallel processing, sensitivity, and shortened turnaround time demonstrate how much further microfluidic integration can push the diagnostic potential compared to a basic RT-PCR test [77]. In addition, immunoassay-based microfluidic assays can detect HIV-1 using a sandwich immunoassay in as little as 0.11 ng/mL, targeting the p24 antigen [78]. For example, several microfluidic devices have been shown to detect Zika virus in a simple, rapid (<30 min), and cost-effective microfluidic test with a limit of detection of 101 virus particles per microliter [79]. In each case, the use of microfluidic integration has been able to greatly improve the current diagnostic capabilities for RNA-based viruses such as HIV-1, Zika and SARS-CoV-2, across a wide variety of detection methods. The potential to advance our diagnostic capabilities by successfully integrating RNA-based detection methods with microfluidics has been demonstrated. As a result, the interest in the commercialization of these platforms has been rapidly increasing [78,79]. One such example of successful commercialization is found in the RNA-based early-cancer diagnostic field, namely GASTROClear DX [80].

GASTROClear DX is a miRNA-based assay that serves as the world’s first molecular blood test for early detection of gastric cancer (GC) within susceptible populations [80,81]. The release of GASTROClear DX in 2020 allowed for the risk assessment of GC in seemingly healthy asymptomatic people while simultaneously provided a notable accuracy of 87% higher than any other conventionally quantified blood biomarker for GC such as serum pepsinogen, CEA, and CA19-9 tumor markers [80,81]. This test is now CE-marked and Singapore Health Science Authority-approved Class C in vitro diagnostics (IVD) permitting commercial availability in Singapore and the ASEAN region along with registration trials in China and Japan [81]. The development of GASTROClear DX and other novel cancer diagnostic methods such as RNA disruption assays not only highlight the necessity of novel diagnostic means but also the potential of RNA-based systems when they are manufactured at a commercial level. Therefore, microfluidic technology has continued to expand our diagnostic capabilities and should be expected to continue expanding our diagnostic capabilities even further into the future.

### 4.3. Implementation of Microfluidics towards Future Preparedness in RNA-Based Diagnostics

The detection and quantification of RNA-based viruses through microfluidic technology currently holds great promise for future diagnostic use [77]. Significant improvements in the detection speeds, portability, limit of detection, and costs will greatly increase the on-site diagnostic potential of microfluidic-based technology for future use. With greater technological advancements expected in the future, microfluidic technology will continue to evolve and overcome several limitations observed in previous and current microfluidic-based diagnostic tools. The expectation is that, through the continued effort and investment into microfluidic technology, rapid and on-site diagnosis of RNA-based viruses, such as SARS-CoV-2, will be more readily accessible to individuals all around the world [82].

Most of the current RNA-based detection methods, shown in the recent literature, demonstrate the numerous benefits of this technology, such as the higher modularity, increased limit of detection, and test turnaround times [73,74,75,76,77]. However, these advancements were not achieved without overcoming some inherent limitations to RNA-based detection, such as the instability and innate reactiveness of exposed RNA in solution [40,42,81,83]. The 2′ hydroxyl group found in RNA increases the likeliness of this type of genetic material to be more prone to dehydration and degradation, therefore, making it a historically poor target for genetic probing in vivo, as many DNA and RNA nucleases exist in commonly used diagnostic sample mediums such as blood, saliva, and nasopharyngeal liquid [22,23,24,25]. Despite this inherent limitation to RNA-based detection methods, several groups have been able to prove its viability and increased diagnostic potential as a useful detection target. For instance, in 2023, Prakash et al. proposed a loop-mediated isothermal amplification (LAMP) method for the detection of SARS-CoV-2 RNA [84]. This LAMP method utilizes RNA extraction from blood samples through the TRIzol method, which amplifies a gene in the form of dsDNA through a single-step reaction with a mixture of incubated primers; a reaction contains dNTPs, HNB (Hydroxy Naphthol Blue), Betaine, MgSO_4_, Bst DNA polymerase, and the target substrate [84]. This preparation step allows the normally reactive single-stranded viral RNA to be paired into a complimentary DNA (cDNA) phase, which in turn, facilitates downstream processing by creating synthetic DNA strands that are far less susceptible to degradation and dehydration, compared to RNA [84,85]. Another diagnostic method that showcases the variety in the stabilization and usage of RNA is the early diagnosis of hepatocellular carcinoma. While prior methods were centered around electrochemical methods such as the biosensor based on PtPd nanodendrite/nano-flowerlike@GO signal amplification for the detection of lncRNA in serum, a more unique RNA detection-based method was recently proposed [85]. Yao et al. proposed a rolling ring amplification (RCA) in which IncRNA was utilized as a carrier for the combination of multiple primers that can then cause RCA reactions [85]. The molecular beacons, in conjunction with the fluorescence signal in the presence of IncRNA, further demonstrate the diagnostic capabilities of IncRNA and its application to RNA detection in human samples [85]. In short, the issues that were historically caused by the instability and reactivity of viral RNA have been successfully circumvented with techniques such as LAMP and RT-PCR. Therefore, it would stand to reason that further integration with microfluidic technology might advance the diagnostic potential of RNA-based methods beyond our current means.

Techniques such as microflow cytometry and nanoparticle-based platforms may offer improved detection limits, sample volumes, and quantitative capabilities when combined with an RNA-based detection method. Currently, integrated microfluidic devices must strive to adhere to the ASSURED standards set out by the WHO and the seven pillars of microfluidic integration, set out by Escobar et al., to the best of their ability [1,85]. Thus, providing a roadmap of properties and functions should be prioritized in the future development of any idealized on-site microfluidic diagnostic device. Given the current state of our diagnostic capabilities, it would be best to set a collection of metrics by which future on-site diagnostic tools can be critiqued upon. Based on the criteria, a minimum limit of detection of 0.20 ng/mL, turnaround times of less than 30 min, sample volumes averaging around 15 µL/test, portability, and some degree of automation can serve as a future minimum standard for the optimized development of future microfluidic diagnostic tools [1]. By setting this base standard of performance metrics, the hope is that industry and researchers alike would be able to focus on more effectively optimizing their designs for the betterment of society and for improved future preparedness.

## 5. Conclusions

Currently, limitations in our rapid and on-site diagnostic capabilities are fewer than they have ever been. However, the poor portability, dependency on instrumentation, and logistical costs associated with mass production and mass distribution reduce the practicality of both our microfluidic and non-microfluidic diagnostic tools. Throughout the COVID-19 pandemic, there was a lack of efficient and reliable diagnostic processes which may be one of the reasons that the illness spread so easily. To better prepare for future pandemics, one must determine how to diagnose the illness as quickly as possible to start treatment and isolation on time. The treatment of viruses also must be considered under developing countries’ conditions because diagnostic techniques that require large expensive equipment may not be possible. Microfluidic devices can combat these issues as they are a rapid, cheap, and easy to use design, attributes which facilitate diagnostic processes. Indeed, they may be able to move along the diagnosis and rehabilitation processes which will in turn lower the spread of disease and death rate. These devices only require a small amount of reagent and greatly reduce the space, effort, and time needed for diagnoses. There have been multiple methods that have been studied within microfluidic devices and each one comes with a unique set of advantages.

Several other examples of RNA-based detection methods have been previously shown to have improved potential in diagnosing viral diseases, other than SARS-CoV-2, through their integration with other analysis methods. Several reports had successfully circumvented the issues inherent to using RNA as a genetic target, using RT-PCR and LAMP, to create cDNA copies of the target RNA and facilitating downstream detection with genetic probes. For example, there was a successful commercialization of an early-stage cancer diagnostic method using RNA-based detection in whole blood and capable of detecting cancer in seemingly asymptomatic patients. In addition, given the current state of microfluidic technology and its recent significant advances in diagnostic applications, microfluidic integration should be considered as a strong candidate for future improvements to our rapid and on-site diagnostic capabilities.

Through this review, we have considered the significant potential for improved future on-site diagnostic potential that microfluidic technology has demonstrated through the lens of past and present advancements in microfluidic technology. The limitations inherent to non-microfluidic devices would not be capable of the diagnostic feats that integrated microfluidic devices can achieve, such as <30 min turnaround times and limits of detection lower than 50 ng/mL. As a result, integrated microfluidic-based diagnostic platforms have the potential to screen for and analyze patient samples in a more realistic setting without loss to accuracy or robustness. Recent advances in this continuously expanding field show why microfluidic technology should continue to be investigated as a strong candidate for the future gold standard of viral diagnostics. Nevertheless, the key obstacles in facilitating this goal such as costs of mass production and mass distribution, as set out by the criteria explored in this review, remain. The improvements and ease of access envisioned in the detection and quantification of several infectious pathogens can be feasibly achieved through continued innovation in microfluidic technology. Therefore, by combining the RNA-based detection methods currently at our disposal with microfluidic platforms and learning from our previous societal and logistical shortcomings from the COVID-19 pandemic, the management of potential future potential viral outbreaks may be significantly improved upon; hopefully reducing the number of deaths and long-term symptomatic infections.

## Figures and Tables

**Table 1:** Metrics used to assess effectiveness in microfluidic substrates. Reprinted from [9].

Property	Silicon/glass	Elastomers	Thermoplastics	Hydrogel	Paper
Young’s modulus	130–180/50–09	~0.0005	1.4–4.1	Low	0.0003–0.0025
Common technique for microfabrication	Photolithography	Casting	Thermomoulding	Casting, Photopolymerization	Photolithography, Printing
Smallest channel dimension	<100 nm	<1 µm	~100 nm	~10 µm	~200 µm
Channel Profile	Limited 3D	3D	3D	3D	2D
Multilayer channels	Hard	Easy	Easy	Medium	Easy
Thermostability	Very High	Medium	Medium to High	Low	Medium
Resistance to oxidizer	Excellent	Moderate	Moderate to Good	Low	Low
Solvent compatibility	Very High	Low	Medium to High	Low	Medium
Hydrophobicity	Hydrophilic	Hydrophobic	Hydrophobic	Hydrophilic	Amphiphilic
Surface charge	Very Stable	Not Stable	Stable	N/A	N/A
Permeability to oxygen	<0.01	~500	0.05–5	>1	>1
Optical transparency	No/High	High	Medium to High	Low to Medium	Low

**Table 2:** Comparison of commonly used detection methods for viral diagnostics.

Method	Year Invented	Description	Advantages	Limitations	Applications
ELISA	1971	Measures specific blood antibody concentrations	Highly sensitive, precise, provides reproducible results	Convoluted process, long turnaround times, susceptible to contamination	Blood borne viruses (HBV, HIV, HCV, etc.)
Western Blotting	1979	Protein concentrations are detected in a blood/tissue sample	Low quantities of reagents are required, making it affordable	Highly dependent on the quality of the sample, long turnaround times	HIV
qPCR	1984	Quantifies DNA amplification throughout a reaction cycle	Extremely precise, sensitive, reliable	Extended turnaround times, costly to train staff	SARS-CoV-2
LAMP	2000	Amplifies DNA to the detectable threshold	User-friendly, low costs, high specificity	Low versatility, reliance on spread of virus (indirect detection)	SARS-CoV-2
RPA	2006	Real-time detection through DNA amplification	Low turnaround times, resource-efficient, economic	Unreliable, poor sensitivity	Respiratory viruses (Influenza, SARS-CoV-2, etc.)

**Table 3:** Comparison of various microfluidic detection technologies. Reprinted from [1].

	Immunoassay	RT-PCR	Nanoparticle	Microflow Cytometry
Reagent Consumption	10 µg (in tube)	20 µL (in tube)	Negligible	50 µL (in tube)
Target of Detection	IgG, IgA, IgM	N gene, E gene	Gold-spiked	IgM, IgG
Limit of Detection	0.15 mg/L	1–10 copy per µL	0.08 mg/L	0.06–0.10 mg/L
Total Assay Time	1 h	2 h	2–5 h	30 min
Sample Volume	20 µL	120 µL	1 µL	10 µL
Assay Control	Automated	Manual	Manual	Automated
Cost per Test	~6 (USD)	~4 (USD)	~10 (USD)	~5 (USD)
Quantitative	No	Yes	Yes	Yes
Mobile	Yes	Yes	No	No

## Data Availability

Available upon request.
